# Retraction: Impact of a Probiotic-Fiber Blend on Body Weight, Metabolic Regulation, and Digestive Function in Obese Adults: A Randomized, Placebo-Controlled, Multicentric Trial

**DOI:** 10.7759/cureus.r255

**Published:** 2026-10-21

**Authors:** Manohar KN, Ramshyam Agarwal, Aparana Patange, Pariksha Rao, Gayatri Ganu, Kaynat Khan, Sanjana Sawant

**Affiliations:** 1 Diabetes and Endocrinology, Manipal Hospitals, Bangalore, IND; 2 General Medicine, Lokmanya Medical Research Center, Pune, IND; 3 Medicine, Krishna Vishwa Vidyapeeth Deemed to be University, Karad, IND; 4 Clinical Nutrition and Gut Microbiome, IMPAct4Nutrition, Bangalore, IND; 5 Pharmacology, Mprex Healthcare Pvt. Ltd., Pune, IND; 6 Nutrition, India Association for Parenteral and Enteral Nutrition, Pune, IND; 7 Research and Development, India Association for Parenteral and Enteral Nutrition, Pune, IND

This article has been retracted by the Editor-in-Chief. Following a post-publication inquiry, a review of the participant-level data and analysis records provided by the authors identified irregularities that undermine confidence in the reported results, and several authors had financial relationships with the study sponsor that were not declared.

